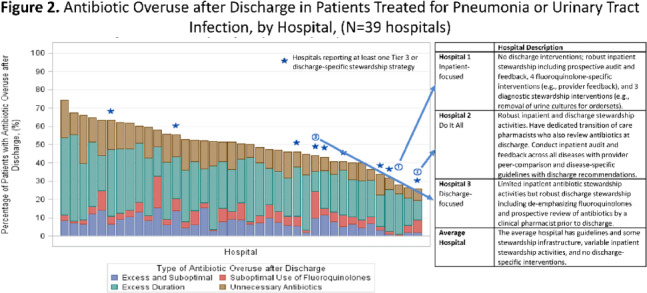# Antibiotic stewardship strategies and antibiotic overuse after hospital discharge: Analysis of the ROAD Home Framework

**DOI:** 10.1017/ash.2022.84

**Published:** 2022-05-16

**Authors:** Valerie Vaughn, David Ratz, M. Todd Greene, Scott Flanders, Tejal Gandhi, Lindsay Petty, Sean Huls, Xiaomei Feng, Andrea White, Adam Hersh

## Abstract

**Background:** Antibiotics are frequently prescribed–and overprescribed–at hospital discharge, leading to adverse-events and patient harm. Our understanding of how to optimize prescribing at discharge is limited. Recently, we published the ROAD (Reducing Overuse of Antibiotics at Discharge) Home Framework, which identified potential strategies to improve antibiotic prescribing at discharge across 3 tiers: Tier 1–Critical infrastructure, Tier 2–Broad inpatient interventions, Tier 3–Discharge-specific strategies. Here, we used the ROAD Home Framework to assess the association of stewardship strategies with antibiotic overuse at discharge and to describe pathways toward improved discharge prescribing. **Methods:** In fall 2019, we surveyed 39 Michigan hospitals on their antibiotic stewardship strategies. For patients hospitalized at participating hospitals July 1, 2017, through July 30, 2019, and treated for community-acquired pneumonia (CAP) and urinary tract infection (UTI), we assessed the association of reported strategies with days of antibiotic overuse at discharge. Days of antibiotic overuse at discharge were defined based on national guidelines and included unnecessary therapy, excess duration, and suboptimal fluoroquinolone use. We evaluated the association of stewardship strategies with days of discharge antibiotic overuse 2 ways: (1) all stewardship strategies were assumed to have equal weight, and (2) strategies weighted using the ROAD Home Framework with tier 3 (discharge-specific) strategies had the highest weight. **Results:** Overall, 39 hospitals with 20,444 patients (56.5% CAP; 43.5% UTI) were included. The survey response rate was 100% (39 of 39). Hospitals reported a median of 12 (IQR, 9–14) of 33 possible stewardship strategies (Fig. 1). On bivariable analyses, review of antibiotics prior to discharge was the only strategy consistently associated with lower antibiotic overuse at discharge (aIRR, 0.543; 95% CI, 0.335–0.878). On multivariable analysis, weighting by ROAD Home tier predicted antibiotic overuse at discharge for both CAP and UTI. For diseases combined, having more weighted strategies was associated with lower antibiotic overuse at discharge (aIRR per weighted intervention, 0.957; 95% CI, 0.927–0.987). Discharge-specific stewardship strategies were associated with a 12.4% relative decrease in antibiotic overuse days at discharge. Based on these findings, 3 pathways emerged to improve antibiotic use at discharge (Fig. 2): inpatient-focused strategies, “doing it all,” and discharge-focused strategies. **Conclusions:** The more stewardship strategies reported, the lower a hospitals’ antibiotic overuse at discharge. However, different pathways to improve discharge antibiotic use exist. Thus, discharge stewardship strategies should be tailored. Specifically, hospitals with limited stewardship resources and infrastructure should consider implementing a discharge-specific strategy straightaway. In contrast, hospitals that already have substantial inpatient infrastructure may benefit from proactively incorporating discharge into their existing strategies.

**Funding:** None

**Disclosures:** None

**Figure:**
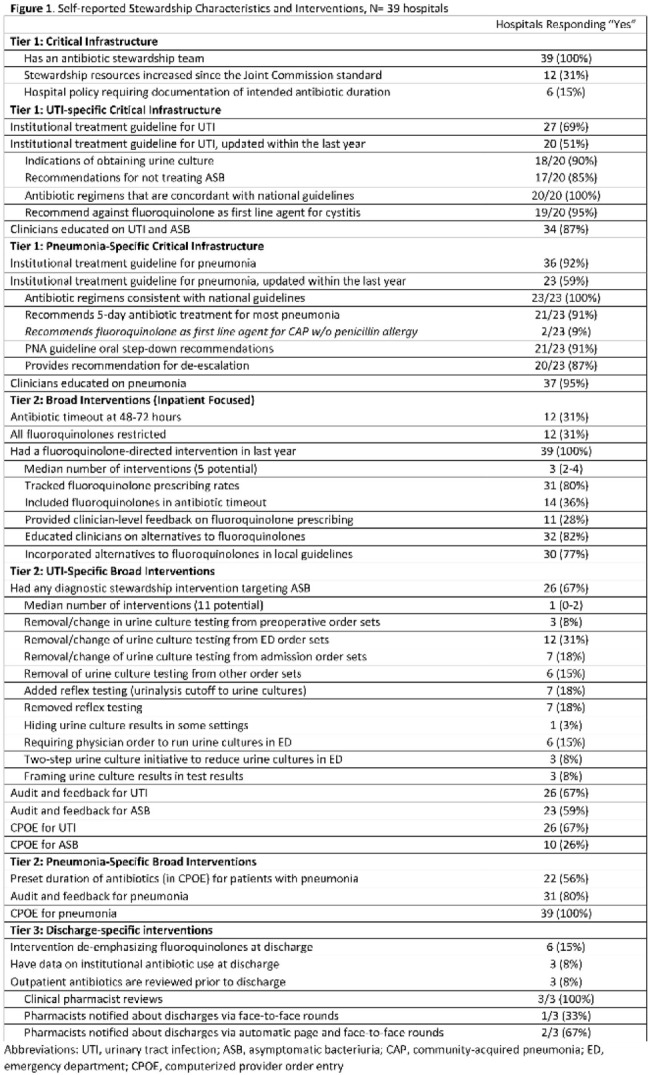

**Figure:**